# Smokeless and combustible tobacco use among 148,944 South Asian adults: a cross-sectional study of South Asia Biobank

**DOI:** 10.1186/s12889-023-17394-w

**Published:** 2023-12-09

**Authors:** Wubin Xie, Malay Kanti Mridha, Anaya Gupta, Dian Kusuma, Awais Muhammad Butt, Mehedi Hasan, Soren Brage, Marie Loh, Khadija Irfan Khawaja, Rajendra Pradeepa, Vinita Jha, Anuradhani Kasturiratne, Prasad Katulanda, Ranjit Mohan Anjana, John C Chambers

**Affiliations:** 1 Population and Global Health, Lee Kong Chian School of Medicine, Nanyang Technological University, Singapore, Singapore; 2 Centre for Non-Communicable Diseases and Nutrition, BRAC James P Grant School of Public Health, Dhaka, Bangladesh; 3 Department of Epidemiology and Biostatistics, School of Public Health, Imperial College London, St Mary’s Campus, Norfolk Place, London, UK; 4 School of Health & Psychological Sciences, City University of London, London, UK; 5 Services Institute of Medical Sciences, Lahore, Punjab Pakistan; 6 MRC Epidemiology Unit, Institute of Metabolic Science, University of Cambridge, Cambridge Biomedical Campus, Cambridge, UK; 7 The Madras Diabetes Research Foundation & Dr. Mohan’s Diabetes Specialties Centre, Chennai, India; 8 Max Helathcare Institute, Patparganj, Delhi India; 9 University of Kelaniya, Sarasavi Mawatha, Sri Lanka; 10 University of Colombo, Mawatha, Colombo, Sri Lanka

**Keywords:** Smokeless tobacco, Smoking behavior, South Asia

## Abstract

**Introduction:**

Tobacco use, in both smoking and smokeless forms, is highly prevalent among South Asian adults. The aims of the study were twofold: (1) describe patterns of SLT and combustible tobacco product use in four South Asian countries stratified by country and sex, and (2) assess the relationships between SLT and smoking intensity, smoking quit attempts, and smoking cessation among South Asian men.

**Methods:**

Data were obtained from South Asia Biobank Study, collected between 2018 and 2022 from 148,944 men and women aged 18 years and above, living in Bangladesh, India, Pakistan, or Sri Lanka. Mixed effects multivariable logistic and linear regression were used to quantify the associations of SLT use with quit attempt, cessation, and intensity.

**Results:**

Among the four South Asian countries, Bangladesh has the highest rates of current smoking (39.9% for male, 0.4% for female) and current SLT use (24.7% for male and 23.4% for female). Among male adults, ever SLT use was associated with a higher odds of smoking cessation in Bangladesh (OR, 2.88; 95% CI, 2.65, 3.13), India (OR, 2.02; 95% CI, 1.63, 2.50), and Sri Lanka (OR, 1.36; 95% CI, 1.14, 1.62). Ever SLT use and current SLT use was associated with lower smoking intensity in all countries.

**Conclusions:**

In this large population-based study of South Asian adults, rates of smoking and SLT use vary widely by country and gender. Men who use SLT products are more likely to abstain from smoking compared with those who do not.

**Supplementary Information:**

The online version contains supplementary material available at 10.1186/s12889-023-17394-w.

## What this paper adds?

• Tobacco use remains a leading risk factor of chronic disease in South Asia. Smokeless tobacco use is particularly popular in this region, even among adult women. Evidence on smokeless tobacco use and smoking cessation are inconclusive and may be dependent on specific cultural and historical factors.

• Contemporaneous data on smokeless and combustible tobacco use patterns in South Asia are limited. Little is known whether SLT use is associated with smoking cessation in South Asia where the vast majority of the world’s SLT users reside.

• This study provided information on patterns of SLT use and smoking in four South Asia countries using a large population-based sample of 148,944 adults collected between 2018 and 2022. We observed that SLT use was associated with higher smoking cessation and lower smoking intensity among men. Strengthening SLT product regulation may have important population health implications with the changing tobacco use landscape in South Asia.

**Supplementary Information:**

The online version contains supplementary material available at 10.1186/s12889-023-17394-w.

## Introduction

Tobacco use accounts for 8.7 million deaths globally and remains one of the leading risk factors of chronic diseases [1]. In South Asia, a large proportion of male adults are current smokers; smoking prevalence is reported to be 46.2% in Bangladesh, 19.1% in India, 21.5% in Pakistan, and 29.4% in Sri Lanka [2, 3]. In contrast, smoking among South Asian females is far less prevalent [3]. Smokeless tobacco (SLT) is used in over 140 countries globally, [4] but is particularly popular in South Asia. India alone is the home to 66.6% of the world’s 356 million SLT users [4]. SLT use is common among women in South Asia, especially in Bangladesh (33%) and India (18.4%).^2,5^ A substantial proportion of south Asian male adults use smokeless and combustible tobacco products concurrently (i.e., dual use) [5, 6]. A recent study found that 9.5% Indian men and 12.5% Bangladeshi men were dual users based on data from Global Adult Tobacco Survey (GATS) 2009 [5]. More recent data from national representative surveys on patterns of SLT and smoking are lacking from South Asia.

Current evidence from developed countries, mostly Sweden, suggests that SLT use is less harmful than smoking, [7, 8] and a comprehensive substitution of smoking with smokeless might result in reduced harm from tobacco use [8]. Thus, at the population level, if SLT products promote smoking cessation a net public health benefit could be expected [9]. However, these assumptions have been questioned in the South Asia context [5, 10, 11]. SLT use has been linked to several types of cancer, [12, 13] cardiovascular disease, [14–17] and adverse birth outcomes when used during pregnancy; [18–21] and the effects vary by geographical regions due to the wide varieties in SLT products and ways of consumption [22]. Moreover, SLT products are known to contain similar or higher nicotine level than cigarettes, [23] and they may be used in situations where smoking is not allowed, possibly prolonging nicotine addiction and making quitting smoking more difficult. The toxicity profiles and health effects of SLT products found in South Asia (often homemade or manufactured by small business) are less clear compared with Swedish snus [8].

Evidence is mixed on whether SLT use facilitates smoking cessation. Studies from Sweden revealed that SLT may be as effective as or even more effective than medicinal nicotine formulations in helping smokers quit [24–26]. A declining smoking prevalence coinciding an increased use of snus was documented in Sweden [27, 28]. However, studies from the US reported that SLT use does not promote smoking cessation, [29, 30] indicating that the relationship may be dependent on specific cultural and historical factors [29]. Limited data are available on SLT use and smoking cessation in the lower- and middle-income countries (LMICs), particularly from South Asia where the vast majority of the world’s SLT users reside [31].

Monitoring patterns of smokeless and smoking patterns and understanding the association of SLT use on smoking intensity, quit attempts, and cessation is important for evaluating the implications of SLT products on population health in this region. Using large-scale population data from the South Asia Biobank (SAB) Study, comprising 148,944 male and female adults from Bangladesh, India, Pakistan, and Sri Lanka, we aimed to (1) describe patterns of SLT and combustible tobacco product use stratified by country and sex, and (2) assess the associations of SLT with attempts to quit smoking, smoking cessation, and smoking intensity among men.

## Methods

### Data source

The SAB is a large comprehensive biobank of South Asian individuals in Bangladesh, India, Pakistan, and Sri Lanka that was established to examine risk factors of diabetes, cardiovascular disease, and other chronic diseases. A detailed description of study design and data collection was previously published [32]. The data from 149,051 adults ages 18 years and above used for the present study were collected between 2018 and 2022. Participants were recruited from 244 surveillance sites that were centered on local primary community health care units in four South Asian countries. To identify resident population, government census data and available household listings were used, together with house-to-house visits by research teams and local primary care workers. The Bangladesh and Sri Lanka samples were drawn from nearly all divisions/provinces/states, India samples were drawn from New Delhi and Chennai, and Pakistan samples were drawn from Punjab.

Residents who were pregnant women, temporary residents (resided for less than 12 month), planned to leave the surveillance site within next 12 months, and had terminal illness were excluded. The overall response rate across all surveillance sites was 67.9% in the early stage of data collection [32]. A rich set of demographic, lifestyle (derived from the WHO STEP questionnaire), clinical, environmental data were collected, along with biological samples. After excluding respondents with missing data on age (n = 2), smoking (n = 12), and with sexual identity of “other” (n = 93), the analytic sample for the present study was 148,944 (Supplementary Figure).

The study was approved by the Imperial College London Research Ethics Committee and also the relevant ethics committees of partner institutes in Bangladesh, India, Pakistan and Sri Lanka. Informed written consent was obtained from all participants.

### Variables

Current SLT use was defined by a yes response to the question, “Do you currently use any snuff, chewing tobacco or betel daily?”. Similar to smoking, ever SLT was determined by a yes response to the question “In the past, did you ever use snuff, chewing tobacco, or betel daily?” or a yes response to the previous question regarding the current use status. Respondents who reported using betel without tobacco were considered non-users. The intensity of SLT use among current users was determined by the question “On average, how many of the following products do you use each day?”. In cases of multiple product use (8.4% of all SLT users), the numbers of each product were added up. The participants were then divided into three categories (0–3, 4–5, 6 + sessions/day).

Current smoking was defined by a yes response to the question “Do you currently smoke any tobacco products daily, such as cigarettes, cigars or pipes?” Non-current smokers were asked whether they ever smoked any tobacco products daily in the past. Those who answered yes to the follow-up question along with who currently smoked were considered ever smokers. An attempt to quit smoking among current smokers was recorded by the question “During the past 12 months, have you tried to stop smoking?”. Smoking cessation was determined if a participant ever smoked any tobacco products daily and currently does not smoke (i.e., a former smoker) and quit smoking for at least one year. Smoking intensity among current smokers was determined by the question, “On average, how many of the following products do you smoke each day?”. In cases of multiple product use (3.7% of all smokers), the numbers of each product were added up.

Based on reported current vs. non-current use of smokeless and combustible tobacco products, respondents were also classified into one of four use patterns: nonuse, exclusive SLT use, exclusive smoking, and dual use. Covariates included age (in years), gender (men, women), and education (no formal schooling, primary school, secondary school, college and above).

### Statistical analysis

We began by conducting descriptive analysis of sociodemographic characteristics, smoking and SLT use patterns stratified by gender and country. All estimates were weighted by poststratification sample weights, which were calculated for each country based on age and sex distribution as estimated by the United Nations Population Prospects 2022 Revision, [33] using STATA IPFWEIGHT procedure [34]. Using an iterative proportional fitting algorithm, first proposed by Deming and Stephan, this approach performs a stepwise adjustment of survey sampling weights to achieve known population margins [34, 35].

Random effects multiple variable-adjusted logistic regression models were used to obtain odds ratios and 95% confidence intervals, quantifying the associations of SLT use with attempting quitting and smoking cessation, adjusting for covariates. Linear mixed models were used to assess the associations between SLT use and smoking intensity among current smokers. Surveillance site was incorporated as a random effect to account for clustering and correlation within geographical locations [36]. All models were adjusted for age, sex, education, and study site, by being incorporated as covariate, or by stratification, or as a random effect. We opted for a conservative covariate adjustment strategy to avoid conditioning on potential mediators, consistent with previous studies [30, 37, 38]. P-values for trends of SLT use intensity were calculated treating SLT use intensity as a continuous variable. Country-specific results were presented given significant heterogeneity by country.

Due to the small number of smokers among the female sample, the analyses assessing the association between SLT use and smoking behavior were restricted to male only. In addition, Participants from Pakistan were excluded from the analysis due to the small number of current SLT users and unstable estimations (for example, among male current smokers, only 24 adults currently using SLT products). While smoking cessation applies to ever smokers, making quitting attempt was applicable to current smokers only.

Exploratory analyses were conducted to assess potential differences by SLT product type (i.e., betel, chew, snuff, and multiple types) via assessing improvement in model fit and similar procedures of testing heterogeneity by country. Analyses were conducted using Stata 17 (STATA Inc.), statistical significance was defined as p < 0.05 (two-tailed).

## Results

Sample characteristics and tobacco use behaviors, stratified by sex and country, are reported in Table 1. On average, participants from Sri Lanka appeared to be older compared with other countries, and participants from India had the highest proportion of adults with a college and above education. Among men, participants from Bangladesh had the highest prevalence of current smoking (39.9%), current SLT use (24.7%), youngest age of smoking initiation (18.9), and highest proportion of attempting to quit smoking in the previous 12 months (55.6%). Bangladeshi women had the highest prevalence of SLT use (23.4%). The mean age of first SLT use tended to be higher than that of smoking. The predominant SLT product type used by current SLT users varied by country. While the most commonly used SLT product in Bangladesh and Sri Lanka was betel, snuff was used by about 70% of Indian adults.

**Table 1:** Sample characteristics and tobacco use behaviours, SAB 2018–2022

Variables ^a^	Men					Women			
	Bangladesh	India	Pakistan	Sri Lanka		Bangladesh	India	Pakistan	Sri Lanka
Number of participants	22,765	12,322	11,914	10,601		27,546	18,730	22,084	22,982
Age in years, mean (sd)	41.9 (16.1)	41.4 (16.1)	38.6 (15.9)	45.2 (17.0)		37.4 (13.6)	40.0 (15.0)	37.4 (14.5)	44.0 (16.1)
Education									
No formal schooling	23.0	6.7	34.1	0.8		25.3	20.2	49.1	1.3
Primary school	17.3	12.4	4.8	7.9		13.8	12.2	4.0	8.2
Secondary/high school	41.3	42.1	28.9	70.7		50.6	38.8	23.2	79.8
College and above	18.5	38.7	32.3	20.6		10.3	29.0	23.7	20.2
Smoking status									
Never	42.9	75.0	87.8	72.8		99.1	98.3	99.5	99.7
Former	17.2	6.4	0.7	12.0		0.5	0.7	0.0	0.1
Current	39.9	18.6	11.5	15.2		0.4	1.0	0.4	0.2
Age started smoke, mean (sd) ^b^	18.9 (6.4)	20.1 (6.3)	23.3 (7.8)	21.4 (6.2)		20.1 (10.8)	24.5 (10.8)	26.8 (11.6)	20.4 (7.1)
Years of smoking, mean (sd) ^b^	22.0 (15.3)	17.6 (14.3)	19.5 (14.1)	21.0 (14.6)		26.2 (17.3)	17.5 (16.4)	22.4 (15.0)	21.7 (16.7)
% Smoking cessation ^b^	29.0	22.7	4.5	42.1		58.4	36.3	4.3	42.8
Smoking intensity, mean (sd) ^c^	8.0 (7.1)	4.7 (6.0)	8.6 (7.6)	3.3 (3.6)		4.3 (3.6)	3.5 (3.9)	5.8 (6.3)	3.2 (2.5)
% Quitting attempt last year ^c^	55.6	48.0	14.8	50.5		55.9	48.5	14.8	58.7
SLT use									
Never	71.3	85.5	98.6	85.5		74.6	98.0	99.9	98.0
Former	3.9	2.5	0.1	1.6		2.0	0.6	0.0	0.4
Current	24.7	12.0	1.3	12.9		23.4	1.4	0.0	1.7
Age started SLT use, mean (sd) ^b^	28.9 (14.2)	21.9 (9.1)	21.6 (7.2)	26.7 (11.1)		29.2 (12.7)	25.7 (11.2)	23.9 (12.5)	32.6 (13.4)
Years of SLT use, mean (sd) ^b^	18.7 (14.6)	16.0 (11.5)	11.9 (9.5)	21.6 (16.9)		16.3 (14.7)	14.3 (13.2)	16.7 (11.9)	24.8 (16.7)
SLT type among current users ^c^									
Betel	56.2	4.1	31.5	73.6		84.8	5.1	-- ^c^	68.7
Chew	32.5	17.2	35.6	2.3		10.0	19.5	-- ^c^	1.7
Snuff	0.3	74.0	29.5	16.2		0.2	68.0	-- ^c^	23.5
Multiple SLT products	11.0	4.7	3.4	8.0		5.0	7.3	-- ^c^	6.1
SLT use intensity, mean (sd) ^c^	6.6 (5.6)	4.2 (4.2)	2.0 (2.5)	4.4 (4.7)		6.0 (4.6)	3.2 (3.0)	3.4 (5.7)	3.2 (2.5)

Abbreviations: SLT, smokeless tobacco

^a^Numbers show percentages, unless indicated otherwise. All estimations, other than sample size, were weighted by post-stratification sample weights, calculated for each country based on age and sex distribution as estimated by the United Nations Population Prospects 2022 Revision

^b^Estimated for ever smokeless tobacco users

^c^Estimates for current users; smokeless tobacco product type was suppressed for female adults in Pakistan due to small numbers

The difference by sex in current smoking was substantial: while the proportion of men who were currently smoking ranged from 11.5% in Pakistan to 39.9% in Bangladesh, only 1% or less women were current smokers in the region. In addition, women tended to have an older age of tobacco use initiation.

Table 2 shows patterns of smoking and SLT use, including concurrent use, stratified by sex and country. Substantial differences in patterns of tobacco use across countries were observed. Among men, the prevalence of exclusive smoking (ranged from 11.3% in Pakistan to 31.6% in Bangladesh) tended to be higher than that of exclusive SLT use (ranged from 1.1% in Pakistan to 16.5% in Bangladesh). Bangladeshi men had the highest rate of dual use (8.3%); only 0.2% Pakistani men were dual users. Consistent with the low rates of smoking, exclusive smoking and dual use among women were rare.

**Table 2:** Current smoking and smokeless tobacco use patterns in South Asia, SAB 2018–2022

Variables ^a^	Men					Women			
	Bangladesh	India	Pakistan	Sri Lanka		Bangladesh	India	Pakistan	Sri Lanka
Number of participants	22,765	12,322	11,914	10,601		27,546	18,730	22,084	22,982
Concurrent use patterns									
Non-use of either product	43.6	73.2	87.4	75.2		76.3	97.7	99.6	98.2
Current SLT only	16.5	8.2	1.1	9.6		23.3	1.3	0.0	1.6
Current smoking	31.6	14.7	11.3	11.9		0.3	1.0	0.4	0.1
Dual use	8.3	3.9	0.2	3.3		0.1	0.1	0.0	0.0
Among ever smokers ^b^									
Non-use of either product	17.0	19.1	5.1	33.8		27.9	38.2	5.4	37.3
Current SLT only	13.1	6.6	0.6	10.2		31.9	3.7	0.0	6.7
Current smoking	55.4	58.9	92.7	43.7		27.1	54.3	92.3	42.3
Dual use	14.5	13.1	1.6	12.2		13.1	3.7	2.3	13.7
Among ever SLT users ^c^									
Non-use of either product	8.5	11.7	6.6	8.7		7.7	28.8	9.9	11.0
Current SLT only	57.5	56.5	78.7	65.9		91.8	66.1	57.8	87.5
Current smoking only	5.2	5.4	0.8	0.2		0.0	1.9	0.0	0.1
Dual use	28.8	26.5	14.0	1.7		0.5	3.2	32.3	1.4

Abbreviations: SLT, smokeless tobacco

^a^Numbers show percentages. All estimations, other than sample size, were weighted by post-stratification sample weights, calculated for each country based on age and sex distribution as estimated by the United Nations Population Prospects 2022 Revision

^b^Reported smoking currently or in the past

^c^Reporting using smokeless tobacco currently or in the past

Among adult men who ever smoked, the majority were current exclusive smokers, and the proportions of male adults who may have transitioned from smoking to exclusive SLT were 13.1%, 10.2%, 6.6, and 0.6% in Bangladesh, Sri Lanka, India, and Pakistan, respectively. On the other hand, among those who ever used SLT products, the majority were current exclusive SLT users. Exclusive smoking among ever SLT users, which may indicate transitioning from SLT use to exclusive smoking, seems uncommon in both male and female samples. The highest proportion of exclusive smoking among ever SLT users were found in Indian men (5.4%). It was rare among adults in Pakistan and Sri Lanka.

Table 3 presents the regression results assessing the associations of SLT use with attempt to quit smoking among male current smokers and smoking cessation among male ever smokers. Ever SLT use was associated with higher odds of making a quitting attempt only in India (odds ratio [OR]1.30; 95% CI, 1.03, 1.66). SLT use was associated with higher odds of smoking cessation, and the magnitude of associations varied across countries. Compared with those never used SLT product, ever SLT users had higher odds of smoking cessation in Bangladesh (OR, 2.58; 95% CI, 2.37, 2.81), India (OR, 2.02; 95% CI, 1.63, 2.50), and Sri Lanka (OR, 1.36; 95% CI, 1.14, 1.62). Moreover, higher SLT use intensity was associated with higher odds of smoking cessation, most obviously in Bangladesh: the ORs for 0–3 session/day, 4–5 sessions/day, and 6 + sessions/day were 1.27 (95% CI, 0.86, 1.63), 2.29 (95% CI, 2.00, 2.62), and 3.68 (3.31, 4.09), respectively (p for trend < 0.001).

**Table 3:** Association of current smokeless tobacco use and smoking behaviour among male participants former or currently smoke, SAB 2018–2022

SLT use status ^f^	Quit attempt ^a^			Smoking cessation ^b^	
	**N (%)** ^c^	**OR (95% CI)**		**N (%)** ^c^	**OR (95% CI)**
**Bangladesh**					
Never SLT use	3664 (55.5)	1 [reference]		1971 (19.8)	1 [reference]
Ever SLT use ^d^	1410 (56.0)	1.07 (0.97, 1.18)		2463 (47.6)	2.88 (2.65, 3.13)
Never/former SLT use	3927 (55.8)	1 [reference]		2420 (22.2)	1 [reference]
Current SLT use ^e^	1147 (54.9)	1.01 (0.91, 1.12)		2014 (46.8)	2.71 (2.48, 2.97)
Current, 0–3 sessions/day	239 (55.6)	0.96 (0.79, 1.17)		198 (29.6)	1.27 (1.06, 1.54)
Current, 4–5 sessions/day	385 (57.7)	1.13 (0.96, 1.14)		595 (46.1)	2.29 (2.00, 2.62)
Current, ≥ 6 sessions/day	523 (52.7)	0.96 (0.84, 1.11)		1221 (52.9)	3.68 (3.31, 4.09)
P for trend		0.401			< 0.001
**India**					
Never SLT use	680 (45.0)	1 [reference]		450 (20.5)	1 [reference]
Ever SLT use ^d^	275 (57.0)	1.30 (1.03, 1.66)		271 (28.5)	2.02 (1.63, 2.50)
Never/former SLT use	742 (46.4)	1 [reference]		524 (21.8)	1 [reference]
Current SLT use ^e^	213 (53.8)	1.11 (0.86, 1.44)		197 (25.9)	1.62 (1.29, 1.63)
Current, 0–3 sessions/day	112 (56.7)	1.37 (0.97, 1.92)		67 (19.2)	1.18 (0.86, 1.63)
Current, 4–5 sessions/day	40 (51.9)	1.00 (0.60, 1.67)		58 (36.1)	2.32 (1.58, 3.42)
Current, ≥ 6 sessions/day	61 (50.0)	0.85 (0.57, 1.28)		72 (29.2)	1.84 (1.31, 2.59)
P for trend		0.886			< 0.001
**Sri Lanka**					
Never SLT use	625 (49.2)	1 [reference]		954 (40.4)	1 [reference]
Ever SLT use ^d^	217 (54.6)	1.19 (0.93, 1.52)		400 (46.9)	1.36 (1.14, 1.62)
Never/former SLT use	644 (49.4)	1 [reference]		1033 (41.5)	1 [reference]
Current SLT use ^e^	198 (54.6)	1.21 (0.94, 1.55)		321(44.1)	1.18 (0.98, 1.62)
Current, 0–3 sessions/day	103 (56.1)	1.27 (0.91, 1.77)		89 (29.6)	0.74 (0.56, 0.99)
Current, 4–5 sessions/day	43 (52.7)	1.19 (0.74, 1.93)		96 (54.0)	1.43 (1.03, 1.99)
Current, ≥ 6 sessions/day	52 (52.7)	1.10 (0.72, 1.70)		136 (55.4)	1.73 (1.29, 2.31)
P for trend		0.670			0.001

Abbreviations: SLT, smokeless tobacco; OR, odds ratio

^a^Responded “yes” to the question “During the past 12 months, have you tried to stop smoking? The question is applicable only to current smokers

^b^Reported ever smoked tobacco product, but do not currently smoke, and abstained from smoking 1 or more years

^c^Unweighted number of individuals made quitting attempt (smoking cessation) and weighted row percentages

^d^Reporting using smokeless tobacco currently or in the past

^e^Reported using smokeless tobacco currently. Smokeless tobacco use intensity is derived from answer to the question “on average, how many of the following products do you use each day”. If a respondent currently uses multiple smokeless tobacco products, the numbers are added up. The reference group is “never/former SLT use”

^f^All estimations adjusted for age and education attainment; study site was incorporated as a random effect to account for clustering and dependence within geographical locations. Pakistan sample was excluded from the analysis due to unstable estimation resulting from small number of current smokeless tobacco users

Turning to SLT use and smoking intensity among male current smokers (Table 4), ever and current SLT use was associated with lower smoking intensity in all three countries. Higher SLT use intensity was associated with lower intensity of smoking in Bangladesh (p-value for trend < 0.001) but not observed in India and Sri Lanka. Compared with non-current SLT use, smoking intensity among current SLT users was 1.38 units lower (95% CI, -1.74, -1.03) in Bangladesh, 1.62 units lower (95% CI, -2.33, -0.92) in India, and 0.47 units (95% CI, -0.90, -0.55) lower in Sri Lanka.

**Table 4:** Association of smokeless tobacco use and smoking intensity among adult male participants currently smoke, SAB 2018–2022

SLT use status ^d^	Smoking intensity ^a^	
	**Adjusted mean (95% CI)** ^e^	**β (95% CI)**
**Bangladesh**		
Never SLT use	9.0 (8.3, 9.8)	[reference]
Ever SLT use ^b^	7.9 (7.1, 8.7)	− 1.14 (-1.47, -0.80)
Never/former SLT use	9.0 (8.3, 9.8)	[reference]
Current SLT use ^c^	7.7 (6.9, 8.4)	-1.38 (-1.74, -1.03)
Current, 0–3 sessions/day	8.2 (7.3, 9.2)	-0.81 (-1.50, -0.12)
Current, 4–5 sessions/day	7.5 (6.6, 8.4)	-1.53 (-2.10, -0.96)
Current, ≥ 6 sessions/day	7.5 (6.7, 8.3)	-1.55 (-2.03, -1.06)
P for trend		< 0.001
**India**		
Never SLT use	5.7 (5.2, 6.1)	[reference]
Ever SLT use ^b^	4.6 (3.9, 5.3)	− 1.05 (-1.72, -0.39)
Never/former SLT use	5.7 (5.2, 6.2)	[reference]
Current SLT use ^c^	4.1 (3.3, 4.8)	-1.62 (-2.33, -0.92)
Current, 0–3 sessions/day	4.0 (3.0, 4.9)	-1.74 (-2.67, -0.82)
Current, 4–5 sessions/day	4.2 (2.7, 5.6)	-1.54 (-2.96, -0.12)
Current, ≥ 6 sessions/day	4.2 (3.1, 5.4)	-1.47 (-2.62, -0.32)
P for trend		0.684
**Sri Lanka**		
Never SLT use	3.5 (3.2, 3.9)	[reference]
Ever SLT use ^b^	3.1 (2.6, 3.5)	− 0.48 (-0.90, -0.55)
Never/former SLT use	3.5 (3.2, 3.8)	[reference]
Current SLT use ^c^	3.1 (2.6, 3.5)	-0.47 (-0.90, -0.03)
Current, 0–3 sessions/day	3.1 (2.5, 3.7)	-0.42 (-0.99, 0.15)
Current, 4–5 sessions/day	2.9 (2.1, 3.7)	-0.63 (-1.47, 0.21)
Current, ≥ 6 sessions/day	3.1 (2.4, 3.9)	-0.42 (-1.17, 0.32)
P for trend		0.729

Abbreviations: SLT, smokeless tobacco

^a^Smoking intensity is defined by the average number of combustible tobacco product smoked per day among current smokers

^b^Reporting using smokeless tobacco currently or in the past

^c^Reported using smokeless tobacco currently. Smokeless tobacco use intensity is derived from answer to the question “on average, how many of the following products do you use each day”. If a respondent currently uses multiple smokeless tobacco products, the numbers are added up. The reference group is “never/former SLT use”

^d^All estimations adjusted for age and education attainment; study site was incorporated as a random effect to account for clustering and dependence within geographical locations. Pakistan sample was excluded from the analysis due to unstable estimation resulting from small number of current smokeless tobacco users

^e^Predicted means (95% CI) are computed from predictions of respective fitted models

In a secondary analysis testing differences by SLT product types, we found a significant improvement in the model fit when current SLT users were further divided by SLT product type for predicting smoking cessation and intensity, but not for predicting making quitting (supplementary Table). These relationships differed by country. Preliminary results suggest that the specific SLT products associated with highest smoking cessation rates were chewing tobacco in Bangladesh (OR, 4.12; 95% CI, 3.56, 4.77), and betel in Sri Lanka (OR 1.47; 95% CI, 1.19, 1.81). Compared with using a single SLT product, simultaneous multiple SLT products use was associated with higher odds of smoking cessation in India, and lower smoking intensity in Bangladesh.

## Discussion and conclusion

In our analysis of a contemporary large population-based data of 148,944 South Asian adults, rates of smoking and SLT use varied widely by country and sex. Bangladeshi men and women had the highest rates of current smoking and SLT use. Among adult males, ever SLT use was associated with a higher likelihood of abstaining from smoking. Higher SLT use intensity was associated with higher smoking cessation. Smoking intensity appeared to be lower among SLT users compared with nonusers.

Tobacco use, in both smoking and smokeless forms, is highly prevalent in South Asia and remains a major risk factor for chronic diseases [1]. Dual use of both smoking and SLT represents an important public health challenge, because dual use may be motivated by intentions to reduce or quit smoking or to circumvent smoking prohibition laws and the health effects of dual use could be synergistic [16, 39]. Our results suggested that dual use is rare among female adults and varies widely among male adults across countries, ranging from 0.2% in Pakistan to 8.3% in Bangladesh. Few studies have reported the concurrent use patterns of smoking and SLT use in South Asia. A study based on the GATS 2016–2017 reported a dual use prevalence of 6.3% among male Indian adults [6]. Another study based on the 2009 GATS data reported a dual use prevalence of 12.5% among male Bangladeshi adults [5]. Our estimations appeared to be lower compared with these numbers, which may be explained by a secular decline (as our data were collected between 2018 and 2022), different definition of current use (current use in GATS studies included both current daily and non-daily use, while our study included current daily use only), and sample compositions. The continuous monitoring of these trends is important.

Our results show SLT use is associated with smoking cessation. While little evidence is available from this region, our findings are consistent with several published studies [39]. However, previous studies also indicated that dual users may be less likely to abstain from all tobacco product due to continuous SLT product use [39]. Consistent with this observation, our descriptive results showed that very small proportions of adults were former SLT users, indicating low rates of SLT product cessation among ever SLT users.

A closely related question regarding public health implications of SLT use is whether SLT facilitates a pathway into smoking (i.e., “gateway effect”) [40]. While this question is best examined with longitudinal studies, our cross-sectional data provided some important insights. First, the average age of first SLT use was greater than that of first smoking, and 85% of our sample started smoking at the same time or prior to SLT use. Second, current exclusive smoking among former SLT users was much less common than current exclusive SLT use among former smokers. These findings favored a general tendency of switching from smoking to SLT use rather than the other way around in this sample of South Asian adults.

Taken together, these findings are consistent with the previous population-based studies, showing that SLT use is replacing smoking in many South Asian countries [5, 41]. In Bangladesh and India, for example, exclusive SLT use has increased along with declining smoking rates, while dual use has remained relatively stable, or declined slightly [26]. Despite the findings showing a positive association between SLT use and smoking cessation in the present study, using SLT as a harm reduction approach to tobacco control in South Asia remains uncertain in this context. Several studies have cautioned considering SLT as a safer alternative to smoking in the South Asian context [5, 10, 11]. SLT products found in South Asia are often homemade or manufactured by small business, a virtually unregulated market [22]. These SLT products may contain higher harmful and potentially harmful constituents relative to Swedish snus, [22, 42] and the relative risks of SLT use tended to be higher in South Asia compared with Europe and North America [43]. Coupled with high prevalence, SLT use accounts for a substantial burden of disease in South Asia [44]. Out of a global disease burden of 348,798 deaths and 8,691,827 disability-adjusted life years (DALY) attributable to SLT use, India alone account for 70%, Pakistan for 7% and Bangladesh for 5%.^44^ In addition, SLT as a replacement for smoking is irrelevant for South Asian women, given SLT is the predominant tobacco product among them [45].

Tobacco smoking is among the leading causes of premature death globally [1]. The negative health effects of smoking are numerous to smokers, as well as to those who are exposed to secondhand smoke [13, 46]. The toxicity profiles and health effects of SLT products used in South Asia are less clear compared with Swedish snus, which some reports estimated to be approximately 5% as harmful as cigarettes [7]. The population health implication of the changing tobacco use landscape in South Asia with increased SLT use along with declining smoking prevalence will at least partly dependent on how SLT products are regulated. Careful profiling of harmful and potentially harmful constituents of SLT products, establishing standards for allowable levels of harmful ingredients are pertinent regulatory actions to protect public health, especially for the socioeconomically disadvantaged groups which are disproportionately affected by SLT use [4, 47]. More research is needed to quantify the levels of acute and long-term exposure to tobacco harmful and potentially harmful constituents and the health effects associated with SLT use in this region.

The study provided an important update on SLT and smoking patterns in South Asia, and among the first, we assessed the association between SLT use and smoking intensity and cessation in South Asia. A major strength of the study is the large sample size collected between 2018 and 2022 with standardized measurements across all four South Asian countries. The number of respondents with missing data on study variables was small. Nevertheless, the study has several limitations. First, SAB used a modified version of WHO STEP questionnaire to measure tobacco use behavior. The original questionnaire does not allow identification of nondaily tobacco users, which could have several implications for the interpretation of the results. To begin with, current tobacco use was defined as “daily use”, which may have resulted in lower estimations of smoking and SLT prevalence, including dual use, by excluding non-daily users. Nevertheless, previous studies (e.g., Sreeramareddy & Aye 2021 [48]; Mutti et al. 2016 [49]) have shown that among current tobacco users, current daily use is the predominant pattern in south Aisa. For example, among male current smokers in Bangladesh about 91% were daily users in the 2017 GATS survey; similarly among current smokeless tobacco users, about 94% were daily users in India and Bangladesh. Moreover, nondaily SLT users, albeit a minority among current users, may have different characteristics and patterns of tobacco use. A previous study from Bangladesh showed that daily dual users were more likely than nondaily dual users to report past attempts and future intentions to quit [50]. Second, tobacco use behaviors were self-reported, misclassification was likely unavoidable, which may have led to attenuated associations in regression analysis. Third, as a cross-sectional study with a conservative covariate adjustment strategy, the associations between SLT and smoking behaviors reported are unlikely to represent causal relationships. Further longitudinal studies are needed to confirm and elaborate these findings. Fourth, the SAB samples were not nationally representative, especially the India and Pakistan samples were drawn from limited number of locations, the findings may not apply to all adults in these countries.

In this large population-based study of South Asian adults, rates of smoking and SLT use vary widely by country and gender. Men who use SLT products were more likely to attempt quitting smoking and to abstain from smoking compared with those who do not, and cessation from smoking was positively associated with SLT use intensity. Continuous monitoring of patterns of SLT use and tobacco smoking is necessary. Given the potential health risks associated with SLT use, strengthening SLT products regulation and promoting SLT cessation is important to protecting public health in South Asia.

### Electronic supplementary material

Below is the link to the electronic supplementary material.

**Supplementary Material 1**: Supplementary Figure. Selection of study participants from South Asia Biobank. Supplementary Table. Association of current smokeless tobacco use and smoking behaviour among male participants former or currently smoke, SAB 2018–2022

## Data Availability

The South Asia Biobank data are available to researchers upon request to the study Data Access Committee. Contact forms and emails are provided on the GHRU website (www.ghru-southasia.org).
