# Supplementary material for: Smokeless and combustible tobacco use among 148,944 South Asian adults: a cross-sectional study of South Asia Biobank
Source: BMC Public Health. 2023 Dec 9;23:2465. doi: 10.1186/s12889-023-17394-w (PMC10709928; doi:10.1186/s12889-023-17394-w)
Supplement: Supplementary file 1 — Supplementary Material 1: Supplementary Figure. Selection of study participants from South Asia Biobank. Supplementary Table. Association of current smokeless tobacco use and smoking behaviour among male participants former or currently smoke, SAB 2018–2022 [file 12889_2023_17394_MOESM1_ESM.docx]

**Supplementary Materials**

**TITLE:** Smokeless and Combustible Tobacco Use Among 148,944 South Asian Adults: A Cross-sectional Study of South Asia Biobank

**AUTHORS:** Wubin Xie;^1^ Malay Kanti Mridha;^2^ Anaya Gupta;^1,3^ Dian Kusuma;^4^ Awais Muhammad Butt;^5^ Mehedi Hasan;^2^ Soren Brage;^6^ Marie Loh;^1,3^ Khadija Irfan Khawaja;^5^ Rajendra Pradeepa;^7^ Vinita Jha;^8^ Anuradhani Kasturiratne;^9^ Prasad Katulanda;^10^ Ranjit Mohan Anjana;^7^ John C Chambers^1,3^

**AFFLIATIONS**

^1^ Population and Global Health, Lee Kong Chian School of Medicine, Nanyang Technological University, Singapore

^2^ Centre for Non-communicable Diseases and Nutrition, BRAC James P Grant School of Public Health, Dhaka, Bangladesh

^3^ Department of Epidemiology and Biostatistics, School of Public Health, Imperial College London, St Mary’s Campus, Norfolk Place, London, UK

^4^ School of Health & Psychological Sciences, City University of London, London, UK

^5^ Services Institute of Medical Sciences, Lahore, Punjab, Pakistan

^6^ MRC Epidemiology Unit, Institute of Metabolic Science, University of Cambridge, Cambridge Biomedical Campus, Cambridge, U.K.

^7^ The Madras Diabetes Research Foundation & Dr. Mohan’s Diabetes Specialties Centre, Chennai, India

^8^ Max Healthcare Institute, Patparganj, Delhi, India

^9^ University of Kelaniya, Sarasavi Mawatha, Sri Lanka

^10^ University of Colombo, Mawatha, Colombo, Sri Lanka

**ADDRESS FOR CORRESPONDENCE**

Wubin Xie, DrPH

Lee Kong Chian School of Medicine, Nanyang Technological University, Singapore.

Tel: +65 65138572

Email: wxie@gwu.edu

**Supplementary Figure. Selection of study participants from South Asia Biobank**

Adults ages 18 years and above

(n = 149 051)

- Sexual identify as “other” (n=93)
- Missing data on age (n=2)
- Missing data on smoking status (n=12)

Adults with pertinent information (n = 148 944)

**Adult female Sample**

(n = 91 342)

**Adult male Sample**

(n = 57 602)

- Pakistan sample (n=11914)
- Never smokers (n=26 029)

**Adult male ever smoked**

(n = 19 659)

- Former smokers (n=6752)

**Adult male currently smoke**

(n = 12 907)

| **SLT type** ^e^ | **Smoking cessation ^a^** | |  | **Intensity ^b^** | |
| --- | --- | --- | --- | --- | --- |
|  | **N (%)** ^c^ | **OR (95% CI)** |  | **Adjusted mean**  **(95% CI)** ^e^ | **β**  **(95% CI)** |
| **Bangladesh** |  |  |  |  |  |
| Never/former SLT use | 2420 (22.2) | 1 [reference] |  | 9.0 (8.4, 9.7) | 1 [reference] |
| Current SLT use ^d^ |  |  |  |  |  |
| Snuff | 2 (14.0) | 0.67 (0.14, 3.15) |  | 11.7 (7.4, 15.9) | 2.61 (-1.56, 6.79) |
| Betel | 1194 (43.2) | 2.22 (2.01, 2.46) |  | 8.4 (7.6, 9.2) | -0.64 (-1.06, -0.22) |
| Chew | 585 (55.6) | 4.12 (3.56, 4.77) |  | 6.0 (5.1, 7.0) | -3.00 (-3.69, -2.31) |
| Multiple products | 230 (48.1) | 3.38 (2.73, 4.19) |  | 5.7 (4.5, 6.8) | -3.39 (-4.39, -2.39) |
| **India** |  |  |  |  |  |
| Never/former SLT use | 524 (21.8) | 1 [reference] |  | 5.7 (5.2, 6.2) | 1 [reference] |
| Current SLT use ^d^ |  |  |  |  |  |
| Snuff | 123 (24.2) | 1.49 (1.15, 1.94) |  | 3.8 (3.0, 4.7) | -1.87 (-2.67, -1.07) |
| Betel | 11 (30.8) | 1.67 (0.76, 3.66) |  | 6.5 (4.0, 9.0) | 0.79 (-1.72, 3.29) |
| Chew | 48 (28.1) | 1.81 (1.19, 2.75) |  | 3.6 (2.2, 5.1) | -2.06 (-3.53, -0.60) |
| Multiple products | 15 (38.6) | 2.96 (1.37, 6.41) |  | 6.5 (3.6, 9.4) | 0.80 (-2.11, 3.70) |
| **Sri Lanka** |  |  |  |  |  |
| Never/former SLT use | 1033 (41.5) | 1 [reference] |  | 3.5 (3.2, 3.8) | 1 [reference] |
| Current SLT use ^d^ |  |  |  |  |  |
| Snuff | 29 (28.0) | 0.73 (0.45, 1.18) |  | 3.1 (2.2, 4.0) | -0.44 (-1.36, 0.48) |
| Betel | 270 (50.2) | 1.47 (1.19, 1.81) |  | 3.1 (2.6, 3.6) | -0.45 (-0.97, 0.66) |
| Chew | 6 (41.0) | 0.94 (0.31, 1.18) |  | 4.3 (1.8, 6.9) | 0.79 (-1.75, 3.34) |
| Multiple products | 11 (20.5) | 0.37 (0.18, 0.76) |  | 3.1 (1.9, 4.2) | -0.48 (-1.67, 0.70) |
| Abbreviations: SLT, smokeless tobacco; OR, odds ratio.   1. Reported ever smoked tobacco product, but do not currently smoke, and abstained from smoking 1 or more years. 2. Smoking intensity is defined by the average number of combustible tobacco product smoked per day among current smokers. 3. Unweighted number and weighted prevalence by categories. 4. Reported using smokeless tobacco currently. 5. All estimations adjusted for age and education attainment; study site was incorporated as a random effect to account for clustering and dependence within geographical locations. Pakistan sample was excluded from the analysis due to unstable estimation resulting from small number of current smokeless tobacco users. | | | | | |

**Supplementary Table. Association of current smokeless tobacco use and smoking behaviour among male participants former or currently smoke, SAB 2018 - 2022**
